# GVHD after CAR T-cell therapy post allogeneic hematopoietic cell transplantation — successfully treated by extracorporeal photopheresis

**DOI:** 10.3389/fimmu.2024.1500177

**Published:** 2024-11-18

**Authors:** Kiavasch Mohammad Nejad Farid, Gesine Bug, Anita Schmitt, Fabian Lang, Maria-Luisa Schubert, Uwe Haberkorn, Carsten Müller-Tidow, Peter Dreger, Michael Schmitt

**Affiliations:** ^1^ Internal Medicine V, Hematology, Oncology and Rheumatology, Heidelberg University Hospital, Heidelberg, Germany; ^2^ Department of Medicine 2, Hematology and Oncology, Goethe University Frankfurt, University Hospital, Frankfurt, Germany; ^3^ Department of Nuclear Medicine, University Hospital Heidelberg, Heidelberg, Germany

**Keywords:** graft-versus-host disease, CAR T-cell therapy, extracorporeal photopheresis, acute lymphoblastic leukemia, T-cell alloreactivity

## Abstract

**Introduction:**

CAR T-cell therapy is highly effective, but also associated with unique toxicities. Because of the origin of T cells in patients who previously underwent allogeneic hematopoietic cell transplantation (alloHCT), graft-versus-host disease (GVHD) in the post-CAR T-cell setting poses a relevant concern but is only scarcely studied. Potential risk factors and mitigation strategies (from CAR T-cell modifications to clinical management) are yet to be determined.

**Methods:**

Sharing our retrospective experience and a mini-review of the literature, our aim is to better understand the frequency and risk of the potential occurrence of GVHD after CAR T cells, which are most likely underestimated.

**Results:**

Here, we present a cohort of 11 patients with symptoms suggestive of GVHD out of 25 allografted patients treated with CAR T cells, of whom 3 patients (12%) had GVHD most likely triggered by the preceding CAR T-cell treatment. Severe chronic pulmonary GVHD occurred in a patient after CD19-directed CAR T-cell therapy. Extracorporeal photopheresis (ECP) mediated successful long-term control of GVHD without causing relapse of the underlying disease.

**Discussion/Conclusion:**

In conclusion, CD19-directed CAR T-cell therapy seems to be feasible in patients after alloHCT but might comprise the potential risk of triggering GVHD, most likely depending on the T-cell source, donor compatibility, and the specific CAR construct used.

## Introduction

CAR T-cell therapy has emerged as a revolutionary treatment option for relapsed or (chemo-)refractory (R/R) hematological malignancies, particularly B-cell precursor (BCP) ALL, (B-cell-)non-Hodgkin lymphomas (NHLs), or multiple myeloma. While commercial CAR T-cell products have found their way up to the second treatment line in some NHL entities, the frontline consolidation strategy in high-risk ALL and the treatment of R/R ALL remain to be allogeneic HCT (1). Unfortunately, a substantial proportion of these patients relapse after HCT. In these cases, CAR T cells are also used in allogeneic HCT recipients. Besides cytokine release syndrome (CRS), immune effector cell-associated neurotoxicity syndrome (ICANS), and immune effector cell-associated hematotoxicity (ICAHT) as typical and unique complications (2, 3), the application of T cells of donor origin harbors the risk of exacerbating preexisting graft-versus-host disease (GVHD) or induces *de novo* GVHD independent of apheresis source and whether manipulated with a CAR construct or not. The infusion of unmanipulated T cells as donor lymphocyte infusions (DLIs) has a longstanding history and is an effective method to enhance graft-versus-tumor [e.g., graft-versus-leukemia (GVL)] effects, while accepting the risk of potential GVHD. By nature, T cells for the production of CAR T cells also possess GVH alloreactivity — they can be collected from the original stem cell donor (true allogeneic, donor-derived CAR T cells) or from the patient/recipient. The T-cell source, the CAR itself, and the used costimulatory domains are thought to contribute to different degrees of alloreactivity and GVHD risk (4–7).

At our center, we currently treat more than 60 patients annually with all commercially available CAR T-cell products and have treated over 220 patients since 2018. Nonetheless, the second-most used CAR T-cell construct is our in-house produced third-generation CAR used in our HD-CAR-1 trial (8) (Eudra-CT no. 2016-0048; NCT03676504), which has shown great responses alongside excellent tolerability. Altogether, we observed very few cases of probable/proven GVHD or possible GVHD-like phenomena after CAR T-cell therapy in allogeneic HCT recipients. Here, we describe a patient with *BCR::ABL1*-positive BCP ALL who developed severe cGVHD post-CAR T-cell therapy, systematically review our own experience with CAR T-cell-triggered GVHD, and summarize the literature on this niche topic in the rapidly growing field of CAR T-cell therapy. This work focuses on the use of CD19-directed CAR T cells in the post-transplant setting (mostly in patients with BCP ALL).

## Methods

Standard information systems *pubmed.org* and *Google Scholar* were screened for keywords “CAR T-cell therapy” and (written as AND) “GVHD” as of 21 January 2024. Furthermore, abstracts from recent international conferences such as the American Society of Hematology (ASH) Annual Meeting in December 2023 and the European Society for Blood and Marrow Transplantation (EBMT)–European Hematology Association (EHA)–CAR Conference in February 2024 were analyzed. All data of the patients treated at our institution (October 2018–October 2021) were obtained by electronic chart review. All patients gave written informed consent to the scientific use of their data and the publication of these data.

Ethical approval and approvals from the local and federal competent authorities were granted as for previous related works (8, 9). HD-CAR-1 trial protocol received Institutional Review Board approval from the EC of the University of Heidelberg in October 2017 (AFmu-405/2017). The present study was performed in accordance with the ethical standards as laid down in the 1964 Declaration of Helsinki and its later amendments or comparable ethical standards.

## Results

Of all 25 allografted patients (NHL, *n* = 8 and acute leukemia, *n* = 17) in our cohort treated with our in-house produced third-generation CAR T cells (recipient-derived; including both costimulatory domains 4-1BB and CD28), 11 patients had symptoms suggestive of GVHD. Of the 17 acute leukemia patients [16 ALL and 1 mixed phenotype acute leukemia (MPAL)], 7 developed signs of acute GVHD (aGVHD) and 1 developed signs of chronic GVHD (cGVHD). In four cases (one NHL patient and three ALL patients), symptoms could be attributed to another more likely etiology (other than GVHD) retrospectively (detailed in **Table 1**). Two of the 25 patients developed cGVHD, one with mucocutaneous involvement and one with severe pulmonary involvement following 4 weeks after CAR T-cell infusion, but following DLIs prior to CAR T-cell therapy. Five patients developed aGVHD (all five patients with gastrointestinal involvement grade I–IV and one patient with cutaneous involvement grade I). Two of those five patients had received a second allogeneic HCT after CAR T-cell therapy, most likely contributing to the development of aGVHD. Three developed true *de novo* GVHD with highly probable association with the preceding CAR T-cell infusions (3/25 = 12%) without any other more probable explanation of symptoms, highly suggestive CAR-to-GVHD time, and no DLI in temporal proximity to the CAR T administration. In all five patients, GVHD occurred approximately 4 weeks after CAR T-cell treatment. Only the higher-grade gastrointestinal GVHDs were histologically proven, while the others were suspected cases (or retrospectively rated as potential GVHD-like phenomena). Of 11 patients with symptoms suggestive of GVHD, 10 were transplanted after total body irradiation-based conditioning (6–12 Gray)/fludarabine and 1 (patient 10, **Table 1**) received treosulfan/fludarabine. All patients with HLA-matched unrelated donors (MUD)/or mismatched unrelated donors (MMUD) received anti-thymocyte globulin (ATG)-based GVHD prophylaxis + calcineurin inhibitor with methotrexate or mycophenolate, and the patient with a matched sibling donor (MSD) (patient 10) did not receive ATG. Full patient characteristics are depicted in **Table 1**.

**Table 1:** Patient characteristics of the allografted cohort with GVHD symptoms.

Patient no.	Age and sex	CART product	Time between HCT and (1st) CART	Donor type	Disease	Best response after CART	GVHD prior to CART (grade)	Symptoms suggestive of GVHD after CART	Onset after CART	(a/c)GVHD probable/proven (grade)	(GVHD) treatment/response	If no GVHD—other more likely explanation	Remarks	Direct CART-association likely?
1	29 f	HDC1	1 yr 5 mo	MUD	ALL	CR	cGVHD skin/oral/hep (moderate)	Dyspnea, cough	4 wk	*cGVHD proven pulmonary/skin (severe)	pred, ECP, FAM, abatacept → PR	–	*GVHD exacerbation after both CART infusions; received DLIs before CART	**Yes**
2	31 f	HDC1	5 yr 9 mo	MUD	ALL	CR	cGVHD oral/hep (mild)	*Diarrhea, pruritus	4 wk	aGVHD probable GI (mild)	Budesonide → CR	-	*GVHD after 2nd CART	**Yes**
3	31 f	HDC1	12 mo	MUD	ALL	CR	aGVHD oc (grade n/a)	*Skin erythema	3 d	No	None	Drug-associated	*Time to onset makes GVHD unlikely	–
4	67 f	HDC1	7 yr 7 mo	MUD	MPAL	CR	aGVHD GI (mild)	*Oral mucositis	7 d	No	None	Chemotherapy-associated	*Time to onset makes GVHD unlikely	-
5	45 m	HDC1	1 yr 8 mo	MUD	ALL	CR	cGVHD oral/oc/hep (moderate)	*Diarrhea, GI bleeding, skin rash	14 mo	aGVHD proven GI (severe)	pred, tac, MMF, budesonide, ECP → PD	–	*GVHD after 2nd allograft, exitus letalis	–
6	37 m	HDC1	3 yr 7 mo	MUD	ALL	CR	n/a	*Skin hyperpigmentation	n/a	No	None	e.g., phototoxicity	*Spontaneous resolution, not typical GVHD	-
7	36 m	HDC1	1 yr 6 mo	MMUD	ALL	–	a- & cGVHD GI (severe)	Abdominal pain/colitis	4 wk	aGVHD proven GI (severe)	dex → n/a	–	Exitus letalis due to MOF	–
8	32 m	HDC1	9 yr 11 mo	MMUD	ALL	CR	aGVHD GI (mild)	Skin rash, diarrhea/nausea	3–4 wk	aGVHD probable skin/gi (mild)	Spontaneous resolution	-	-	**Yes**
9	70 m	HDC1	5 yr 5 mo	MUD	NHL (MCL)	PR	cGVHD serositis/hep (grade n/a)	Conjunctivitis	4 wk	No	None	Ocular relapse	–	–
10	54 m	HDC1	8 yr 1 mo	MSD	NHL (CLL)	PR	*cGVHD skin/hep (moderate)	*Diarrhea	1,5 yr	aGVHD proven GI (severe)	pred, budesonide, tac → PR	-	*GVHD after 2^nd^ allograft	-
11	59 m	HDC1	1 yr 2 mo	MUD	NHL (MCL)	CR (short-lived)	cGVHD skin (mild, short-lived)	Skin rash, sicca	3–4 wk	cGVHD probable skin/oral/oc (moderate)	pred, sirolimus → CR	–	DLI 2,5 months prior to GVHD onset	–

All third-generation CAR T cells were manufactured from recipient-derived T cells. ALL, acute lymphoblastic leukemia; CART, CAR T-cell (infusion); CLL, chronic lymphocytic leukemia; CR, complete remission; d, day(s); dex, dexamethasone; DLI, donor lymphocyte infusion; ECP, extracorporeal photopheresis; f, female; FAM, fluticasone/azithromycin/montelukast; GI, gastrointestinal; (a/c)GVHD, (acute/chronic) graft-versus-host disease; HDC1, HD-CAR-1 trial product; hep, hepatic; m, male; MCL, mantle cell lymphoma; MMF, mycophenolate mofetil; MMUD, mismatched unrelated donor; mo, month(s); MOF, multiorgan failure; MPAL, mixed phenotype acute leukemia; MSD, matched sibling donor; MUD, matched unrelated donor; n/a, not ascertainable; NHL, non-Hodgkin lymphoma; oc, ocular; PD, progressive disease; PR, partial remission; pred, prednisone; tac, tacrolimus; wk, week(s); yr, year(s).

**Figure 1:**
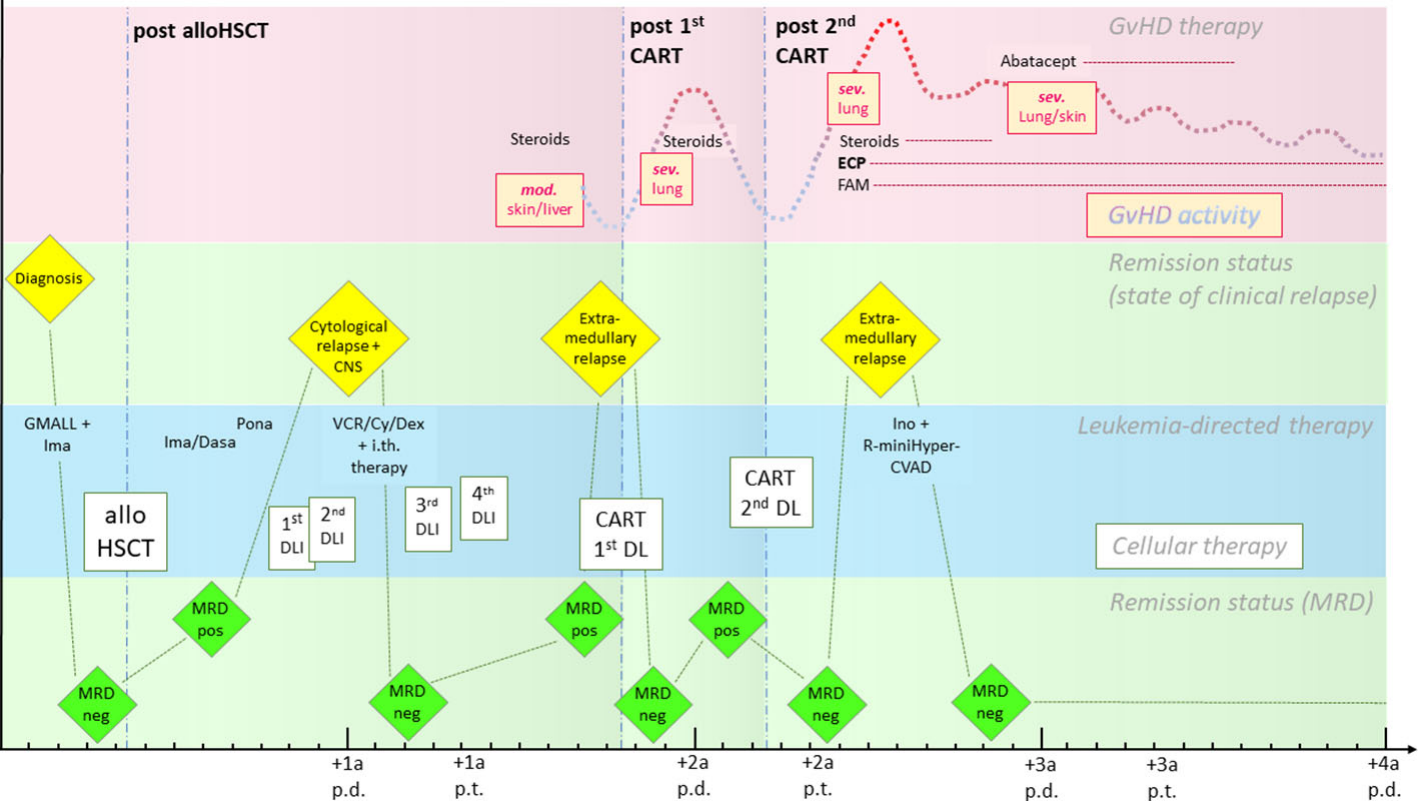
Timeline of the case from diagnosis to last follow-up. The pink row represents the course of GVHD activity (yellow boxes and dotted line) and therapy, the green rows represent remission status (separated into clinical relapse states above and changes in MRD below), and the blue row shows leukemia-directed therapies (cellular therapies are highlighted in boxes). The interval between two minor ticks depicts the duration of a month, between two major ticks a year. CART, CAR T-cell infusion; Cy, cyclophosphamide; Dasa, dasatinib; DL, dose level; DLI, donor lymphocyte infusion; ECP, extracorporeal photopheresis; FAM, fluticasone/azithromycin/montelukast; GVHD, graft-versus-host disease; GMALL, German Multicenter Study Group for Adult ALL; Ima, imatinib; Ino, inotuzumab ozogamicin; i.th., intrathecal; mod, moderate; MRD, minimal residual disease; neg, negative; p.d., post diagnosis; Pona, ponatinib; pos, positive; p.t., post-transplantation; R, rituximab; sev, severe; VCR, vincristine.

Out of the 11 patients described above, we highlight the special case of a 26-year-old woman (patient 1) with Philadelphia chromosome (Ph)-positive BCP ALL (**Figure 1**). She underwent allogeneic hematopoietic stem cell transplantation from a male 10/10 MUD (conditioning: 12 Gray total body irradiation/cyclophosphamide) after achieving molecular remission with intensive induction chemotherapy combined with the tyrosine kinase inhibitor (TKI) imatinib [according to the protocol of the German Multicenter Study Group for Adult ALL (GMALL)]. Shortly after transplantation (+3 months), she suffered a molecular relapse while on imatinib maintenance and was quickly switched to dasatinib followed by ponatinib due to further progression. In the absence of clinically relevant GVHD, immunosuppression was rapidly tapered and two dose-escalated DLIs were given. Despite these measures, the patient developed hematologic relapse with central nervous system involvement (+7 months after HCT). After achieving hematologic remission with systemic and intrathecal chemotherapy again, two more DLIs were given to consolidate remission by reinforcement of the GVL effect. While the immunoglobulin/T-cell receptor gene rearrangement as a minimal residual disease (MRD) marker became negative, the *BCR::ABL1* transcript remained positive, indicating active disease despite maximizing immunological antitumoral effects with the aforementioned methods. Further DLIs were limited by the simultaneous development of cGVHD of the skin and the liver for the first time, treated with systemic steroids. After a total of +16 months after HCT, (isolated) biopsy-proven extramedullary disease was detected in the medial epicondyle of the right arm. She received a first dose (5 × 10^6^ cells/m² body surface area) of third-generation CD19-directed CAR T cells (harvested from the patient and therefore correctly named “autologous”, but *allogeneic* in the strict sense because of donor origin or *“*pseudo-allogeneic”) as part of the HD-CAR-1 trial (8) after lymphodepleting chemotherapy with fludarabine and cyclophosphamide (90 mg/m² and 1,500 mg/m² total dose, respectively). A metabolic remission in the positron emission tomography (PET) scan on day +30 after CAR T-cell infusion and MRD negativity by *BCR::ABL1* polymerase chain reaction in the bone marrow (BM) were observed. In this phase of remission, cough, dyspnea, and peripheral eosinophilia developed alongside worsening of pulmonary function tests, highly suggestive of chronic pulmonary GVHD. Systemic steroids led to rapid resolution of symptoms. Other chronic manifestations of GVHD were treated topically; no other systemic immunosuppressants were necessary. Four months after the CAR T-cell infusion (total +21 months after HCT), molecular relapse was detected in the BM again. A second CAR T-cell infusion was given (escalated dose of 50 × 10^6^ cells/m^2^ body surface area) after lymphodepletion. We observed repeated exacerbation of the pulmonary GVHD, this time with severe functional compromise and symptoms, again requiring steroids. Immunomodulatory therapy with extracorporeal photopheresis (ECP) was initiated. Fluticasone, azithromycin, and montelukast (known as the “FAM” regimen) were also added. Another extramedullary relapse in the maxillary bone again 4 months after the second CAR T-cell infusion (+26 months after HCT) was treated with inotuzumab ozogamicin and chemotherapy (“mini-hyperCVAD” regimen), resulting in complete resolution of the extramedullary disease lesions as well as MRD negativity in the BM. No other chemotherapy or TKI was given thereafter. Severe pulmonary GVHD remained active and cutaneous cGVHD persisted despite ECP and repeated lymphodepleting chemotherapy prior to CAR T-cell infusions. Ultimately, abatacept was added to the immunosuppressive regimen for several months, ameliorating lung function and symptoms. The patient has remained in ongoing molecular remission until today with sufficiently controlled cGVHD activity under ongoing ECP treatment.

**Figure 2:**
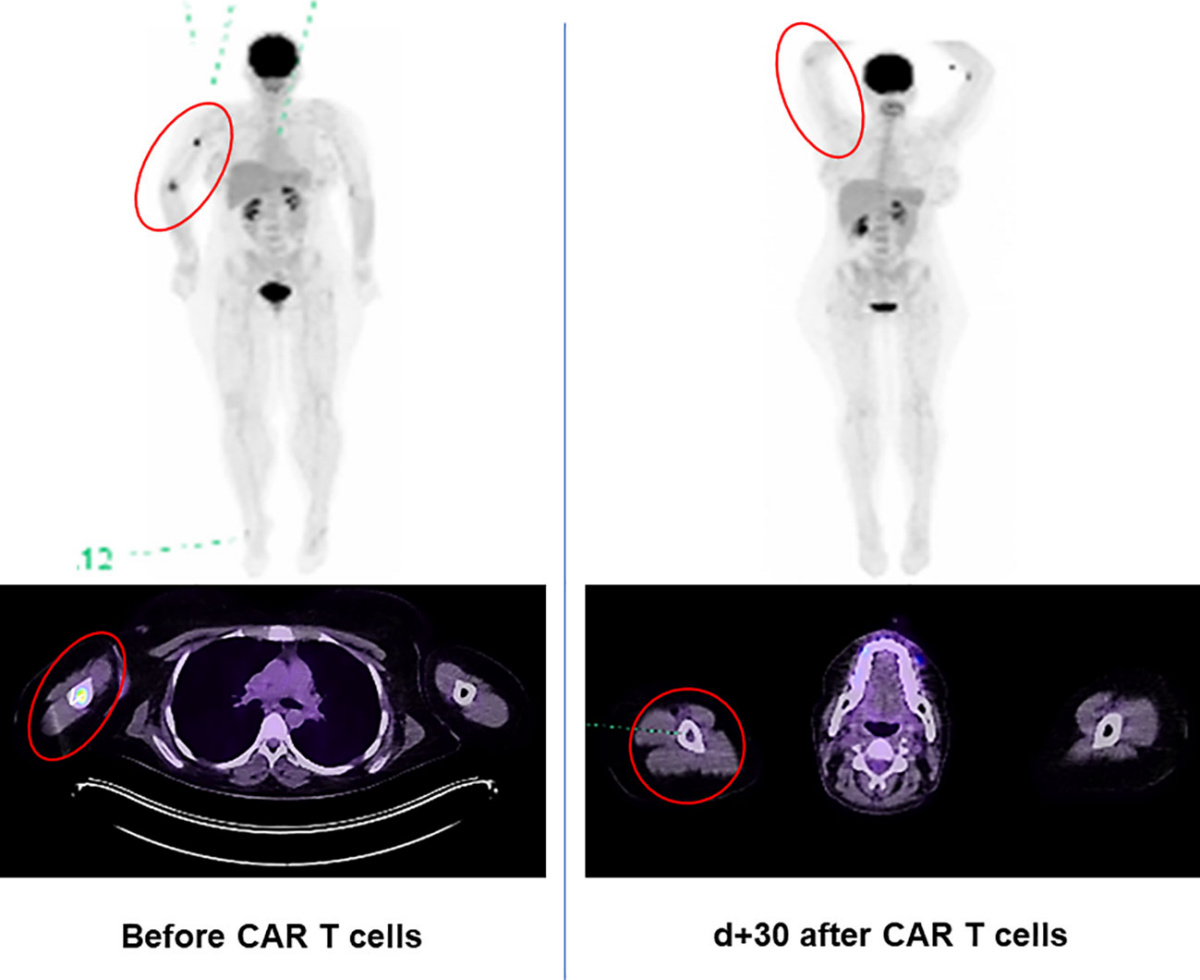
18-Fluordeoxyglucose positron emission tomography (PET) [MIP (maximum intensity projection) and merged transaxial PET/CT] scan of the Ph+ ALL patient with extramedullary relapse in the right medial epicondyle and right humerus. Complete metabolic remission on day 30 after first CAR T-cell infusion (reflected by the decrease in the maximum standardized uptake values from 17.6 to 1.96).

Our patient achieved PET-morphological complete remission (CR) with MRD negativity in the BM early on after both CAR T-cell infusions (**Figure 2**).

**Figure 3:**
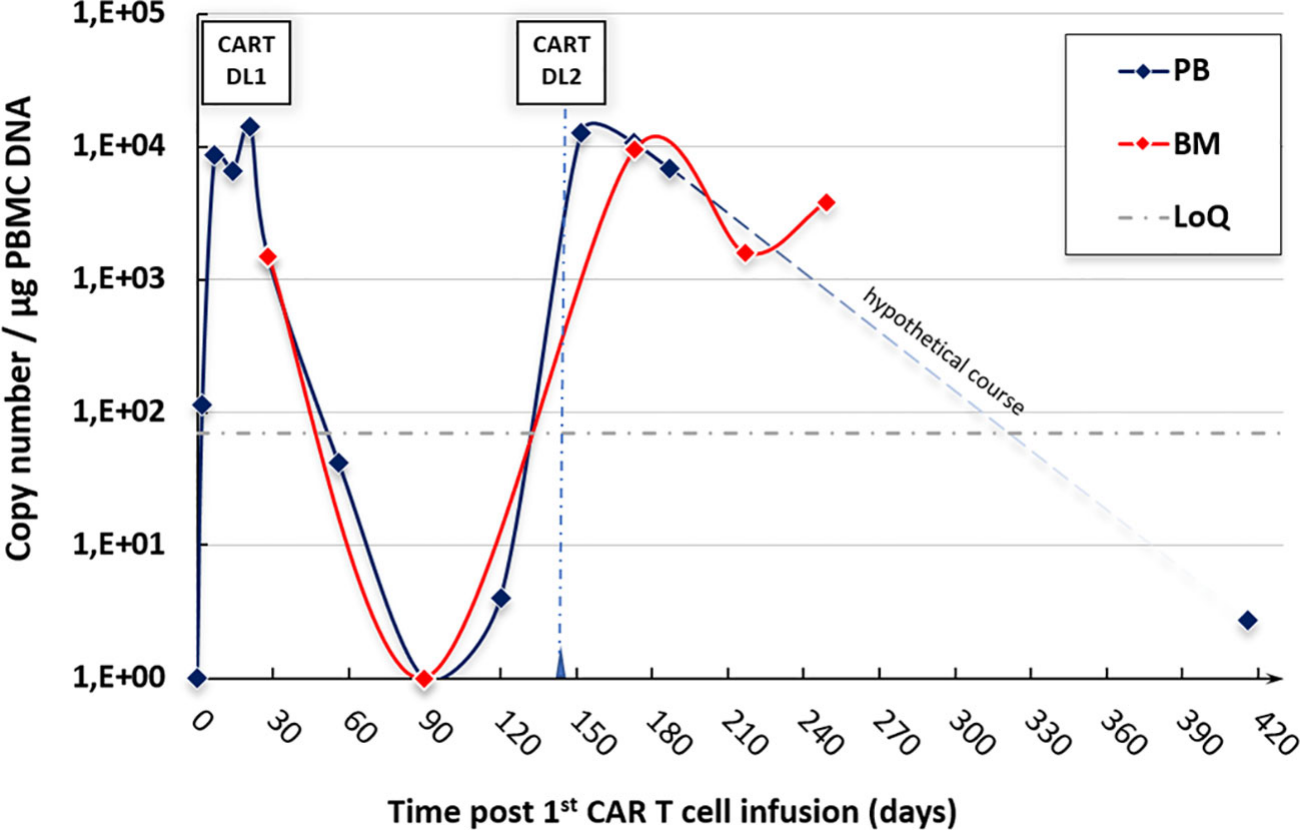
CAR T-cell expansion in our ALL patient after both infusions—rapid expansion after each infusion with detectable persistence >9 months after the second CAR T-cell administration. BM, bone marrow; CART, CAR T-cell infusion; DL, dose level; DNA, deoxyribonucleic acid; LoQ, limit of quantification; PB(MC), peripheral blood (mononuclear cells).

The expansion of the administered CAR T cells in the peripheral blood (and in the BM) is shown in **Figure 3**. Interestingly, the quantitative peak of expansion correlates with the onset of the cGVHD flares as described above. GVHD symptoms exacerbated shortly after the time of the expansion peak, thus suggesting CAR T cells as a trigger of GVHD. Whereas the persistence of CAR T cells was very short-lived after the first infusion, CAR T cells could still be detected over 9 months after the second infusion.

Of note, mild to moderate cGVHD of the skin, oral mucosa, and liver was present in this patient already before the first CAR T-cell infusion. Severe pulmonary involvement [forced expiratory volume in the first second (FEV1) of 33% of the predicted average value and diffusion capacity of carbon monoxide (DLCO) of 33%—grade III] appeared after the first CAR T-cell therapy and could be ameliorated to grade II (FEV1 48%, DLCO 48%) by immunosuppressive therapy (systemic steroids). After the second CAR T-cell infusion, FEV1 decreased to 34% and DLCO decreased to 43% alongside increasing peripheral eosinophilia, making initiation of ECP twice weekly and FAM therapy necessary. Because of steroid-refractory/-dependent GVHD (again grade III—FEV1 29%, DLCO 38%), abatacept was added for a total of over 7 months with ongoing ECP (once weekly). Pulmonary function remained stable ever since, with a FEV1 of 27% and a DLCO of 38% 7 months after the discontinuation of abatacept. Several infections complicated the further course, such as parainfluenza pneumonia, *Pseudomonas aeruginosa* pneumonia, and invasive pulmonary aspergillosis. Notably, the use of leukemia-directed chemotherapy (despite its marked immunosuppressive properties) in the setting of the extramedullary relapse after the second CAR T-cell infusion did not lead to stabilization of pulmonary GVHD, making escalated GVHD-targeted therapy necessary.

To extend our study on GVHD after CD19-directed CAR T-cell therapy, we performed a literature review summarized in **Table 2**, including the seminal review articles by Sanber et al. (7), Smith et al. (6), and Chen et al. (10) A total of 529 patients were included in the studies mentioned, encompassing donor-derived (*n* = 301), recipient-derived (*n* = 128), and *off-the-shelf* CAR T-cell therapies (*n* = 100). Mean incidence of GVHD was 7.6%; 13.9% in donor-derived, 7.8% in the recipient-derived, and 2% in the off-the-shelf CAR T-cell treated cohorts, respectively. In a review by Vittayawacharin et al. (11), CAR T-cell therapy seems to offer a beneficial risk profile regarding GVHD, with no cases of aGVHD and only mild cases of cGVHD appearing after CAR T-cell therapy in non-haploidentical donors. In contrast, Chen et al. observed a high rate of 50% (acute) GVHD in CAR T-cell therapy derived from haploidentical donors (although with a very small patient cohort of 6 patients total) (12). Brudno et al., also cited in the aforementioned reviews, e.g., Smith et al. (6), had shown no GVHD cases in MSDs (13). Hence, the type of donor seems to be relevant for the GVHD risk after CAR T-cell therapy, similar to the stand-alone HCT setting (14). Overall, remission rates of the malignant diseases are roughly comparable between the different studies. The GVHD incidence in patients of the HD-CAR1 trial cohort is comparable to the studies mentioned above.

**Table 2:** List of studies with CD19-directed CAR T-cell therapies and the incidence of GVHD.

Clinical study	No. of patients	T-cell source	Donor type	Costimulatory domain	Disease	Incidence of GVHD (cumulative acute + chronic) in percent
Brudno et al., 2016 (13)	20	d-d	Mixed	CD28	ALL/NHL	10 (only cGVHD)
Kochenderfer et al., 2013 (19)	10	d-d	MSD/MUD	CD28	NHL	None
Chen et al., 2017 (12)	6	d-d	Haplo	n/a	ALL	50 (only aGVHD)
Cruz et al., 2013 (25)	8	d-d	MUD/MMUD	CD28	ALL/NHL	None
Siglin et al., 2020 (18)	6	r-d	Mixed	CD28 (axi-cel)	NHL	50 (only aGVHD; 2/3 haplo)
Del Bufalo et al., 2023 (26)	13	d-d	Mixed	4-1BB	ALL	7.7 (aGVHD)
Luo et al., 2023 (27)	12	d-d	MSD/haplo	CD28	ALL	25 (aGVHD)
Smith et al., 2018 (6) Review (*studies mentioned separately excluded)	27*	r-d	Mixed	CD28	ALL/NHL	None
	56*	r-d	Mixed	4-1BB	ALL/NHL	3.6 (1 aGVHD, 1 cGVHD)
Sanber et al., 2021 (7) Review (*studies mentioned separately excluded)	126*	d-d	Mixed	Mixed	ALL/NHL	16.6 (aGVHD 15, cGVHD 1.6)
	39*	r-d	Mixed	Mixed	ALL/NHL	12.8 (aGVHD 5.1, cGVHD 7.7)
Chen et al., 2022 (10) Review (*studies mentioned separately excluded)	140*	d-d	n/a	Mixed	ALL	2.1
	100	o-t-s	-	4-1BB	ALL/NHL	2

ALL, acute lymphoblastic leukemia; axi-cel, axicabtagene ciloleucel; CD, cluster of differentiation; d-d, donor-derived; (a/c)GVHD, (acute/chronic) graft-versus-host disease; haplo, haploidentical donor; MSD, matched sibling donor; MUD, matched unrelated donor (10/10); MMUD, mismatched unrelated donor (9/10); n/a, not available; NHL, non-Hodgkin lymphoma; o-t-s, off-the-shelf; r-d, recipient-derived.

## Discussion

The use of CAR T cells in allografted patients, especially in ALL patients, harbors unique complications due to complex interactions between graft- and host-derived immune cells in the preexisting state of an immunological chimera. The source of T cells, i.e., recipient- or donor-derived, might contribute to differences in alloreactivity. One might hypothesize that recipient-derived T cells have already gone through a process of host-directed tolerance induction, especially in patients without clinically apparent GVHD and complete donor chimerism (5).

The question remains whether CAR T cells can be safely given in active GVHD and with unchanged efficacy. The use of recipient-derived T cells in the setting of relapse without any (c) GVHD and assumably inadequate GVL effect could be predictive of low T-cell quality/anti-tumoral reactivity. Furthermore, the conditioning platform before alloHCT, and especially the usage of post-transplant cyclophosphamide, could influence the alloreactivity and/or quality of the T-cell subsets used for CAR T-cell manufacturing later on. In our highlighted case, the patient already had signs of cGVHD prior to relapse and CAR T-cell infusion, but also had received several DLIs beforehand. In addition, the subsequent therapies of GVHD and relapse could have a significant impact on the effectivity of CAR T-cell therapy. Immunosuppression (especially T-cell-directed) bears the risk of affecting antitumoral activity (CAR T-versus-tumor), as reflected by the higher relapse probability when the GVL effect is dampened after conventional allografting (15). Thus, we propose the usage of ECP as a potential treatment without hampering the antitumoral activity of previously given CAR T cells (16).

As published before, the use of commercial products such as axicabtagene ciloleucel (axi-cel) for NHL after allogeneic HCT is also feasible, with none of the patients developing GVHD at our institution (17). Surprisingly, another study showed a high aGVHD incidence of 50% after axi-cel, although with limited validity/comparability due to a very small patient cohort including a high proportion of haploidentical donors (18). The use of (non-commercial) donor-derived CAR T cells in NHL patients transplanted from an MSD or MUD showed excellent safety without any occurrence of GVHD in another study (19). However, experience with CAR T-cell therapy in allografted NHL patients is still limited when compared to BCP-ALL patients, since allogeneic HCT is rarely performed prior to CAR T-cell therapy in the era of second-line CAR T-cell therapy option in some NHL subentities (20).

A direct association between the dynamics of CAR T-cell expansion and GVHD risk can be suspected given that an increased GVHD probability was observed (in a murine model) with CAR T-cell products using the 4-1BB costimulatory domain (4), which is known to induce more robust CAR T-cell persistence compared to constructs with other costimulatory domains. The time-dependent effect/risk influenced by the *in vivo* behavior (reflecting the true efficacy) of the (CAR) T cells seems to be similar to the observations known from the use of DLI, where GVL and concomitant GVHD usually appear delayed (21). The use of a CD28-costimulatory domain might be associated with a decreased risk of GVHD due to higher occurrence of activation-induced cell death or accelerated exhaustion (4, 6). Another hypothetical mechanism of CAR-T-triggered GVHD could be indirectly via cytokine-driven activation of preexisting alloreactive T cells. The impact of combining both costimulatory domains within a third-generation CAR constructs and its possible implications for CAR T-cell expansion/persistence on GvH-alloreactivity remains to be elucidated. We report hitherto the first case of severe GVHD (exacerbation) after third-generation CAR T-cell therapy.

In the case described above, the presence of Ph-positive ALL relapse conferred a problem choosing further treatment options. Besides disease progression under imatinib and dasatinib as well as the presence of Q252H mutation, described to be associated with rapid disease progression (22), the use of *BCR::ABL1*-directed TKIs was limited, since TKIs (e.g., dasatinib) are known to hamper CAR T-cell activity (23). Therefore, TKIs were not used with the aim to leave CAR T-cell function as uncompromised as possible. Further DLI administration was not feasible due to active GVHD.

The causal relation between the development of GVHD and the CAR T-cell infusions in the reported case of our patient cannot be determined undoubtedly, since she had received DLIs prior to CAR T-cell therapy and had already developed first signs of cGVHD. Nonetheless, the temporal sequence and latency of GVHD exacerbation is very suggestive of CAR T-cell-induced or -enhanced alloreactivity. Delayed graft-versus-host action of the DLIs (given prior to the CAR T cells) might be attenuated due to possible eradication of those alloreactive T cells by lymphodepletion. From another point of view, there is the hypothetical concern that introduction of CAR T cells into a recipient with depleted lymphocytes might even more so trigger severe GVHD (7, 13), potentially by depletion of tolerance-providing regulatory T cells. More so, in studies of donor-derived CAR T cells, lymphodepletion has been mostly omitted due to the unpredictable risk of GVHD (7), most likely due to facilitated T-cell expansion (as shown for DLI) (24).

Our study has limitations, one of which is its retrospectivity, as well as the fact that not all suspected GVHD cases were proven histologically. The possibility of detection of CAR T-cell-specific gene sequences or CAR-specific immunofluorescence staining in the involved tissue might help to confirm the CAR T-cell-derived origin of GVHD. On the other hand, the patient cohort at our center was well-balanced with allografts mainly from matched donors and use of the same CAR T-cell product.

In conclusion, the risk of GVHD seems to be low when compared to DLI or a second allogeneic graft. In our single-center experience, triggering *de novo* GVHD or exacerbating preexisting GVHD by CAR T-cell therapy is a possible but rather rare complication the treating physician should be aware of. GVH reactivity might occur even when using previously tolerized T cells, regardless of the source of the leukapheresis product. We demonstrate the option of successful long-term control of severe cGVHD after CAR T-cell infusion by ECP. Further studies are needed to mitigate the risk of unselective alloreactivity in cellular immunotherapies, e.g., CAR T-cell therapy.

## Data Availability

The data analyzed in this study is subject to the following licenses/restrictions: Patient’s data obtained by electronic chart review. Requests to access these datasets should be directed to farid.kiavasch@med.uni-heidelberg.de.
